# O4.5. INVESTIGATING GENETIC PROFILES ASSOCIATED WITH ‘REAL WORLD’ CLINICAL OUTCOMES IN PSYCHOSIS: A RETROSPECTIVE COHORT STUDY

**DOI:** 10.1093/schbul/sby015.211

**Published:** 2018-04-01

**Authors:** Emma Francis, Olesya Ajnakina, Marta Di Forti, Robin Murray, Robert Stewart, Conrad Iyegbe

**Affiliations:** 1 South London and Maudsley NHS Foundation Trust; 2 Institute of Psychiatry, Psychology & Neuroscience, King’s College London

## Abstract

**Background:**

The Clinical Record Interactive Search (CRIS) database provides anonymised data from the full electronic health records of all patients at the South London and Maudsley NHS Foundation Trust, a large provider of secondary mental health care. We have previously shown how the large volumes of available CRIS data pertaining to outcomes can be mined and integrated with patient data collected by historical research interview.

The applications of this futuristic translational research model are yet to be fully explored. The aim of this study is to determine whether transcriptomic profiles at the onset of psychosis can discern the likely trajectory symptoms over the subsequent 5 years of illness.

**Methods:**

The study sample consists of 200 first-episode psychosis cases (ICD-10 codes: F20-F29 or F30-F33) aged 18–65 years who presented to SLAM (South London and Maudsley NHS Trust) mental health services between the 1st of January 2010 and the 1st of January 2015. Patients were subsequently recruited to the GAP study. Patients were followed-up electronically for 5 years post recruitment using the CRIS research platform.

RNA samples were collected at the baseline timepoint via PAXgene blood tubes and interrogates, using the Illumina HumanHT-12.v4 beadchip array. Samples were run at the National Institute for Health Research’s (NIHR) Biomedical Research Centre for Mental Health (BRC-MH) at the Institute of Psychiatry, Psychology and Neuroscience. A total of 4756 probes passed a stringent quality control across the 200 samples.

**Results:**

CRIS data pertaining to the GAP cohort was interrogated for information on clinical symptoms over a 5-year period using text-mining and natural language processing apps that represent over 70 different dictionary definitions of psychotic and affective symptoms. Confirmatory factor analysis was used to reduce this to a much smaller set of orthogonal symptom dimensions which were then the subject of a genetic interrogation using gene expression data. The analysis was conducted using a statistical learning framework which combines Elastic net penalised regression methodology with K-fold cross-validation (via the GLMnet package in R). This identified gene transcripts that were predictive of longer term symptom trajectories in half of the available sample. The veracity of the model was further validated using the second withheld portion of the sample.

**Discussion:**

The results of this discovery phase may provide a rationale for subsequent multi-modal investigations whose aims will be to further enrich the biomarker signature and to also understand the molecular mechanisms that sustain them.

